# Gunshot wound without entrance hole: where is the trick? – a case report and review of the literaturer

**DOI:** 10.1186/s13017-015-0048-z

**Published:** 2015-11-04

**Authors:** Silvia Ministrini, Gianluca Baiocchi, Frida Pittiani, Daniele Lomiento, Federico Gheza, Nazario Portolani

**Affiliations:** Surgical Clinic, University of Brescia, P.le Spedali Civili, 1, 25123 Brescia, Italy; Radiologic Department, University of Brescia, Brescia, Italy

**Keywords:** Gunshot, Atypical inlet hole, Intestinal perforation, Diagnosis, Laparotomy, Laparoscopy

## Abstract

The presence at CT scan of more retained bullets than expected could be a very difficult interpretation challenge in the early management of gunshot wounds. The modern non operative management of haemodinamically stable patients without peritonitis requires that the trajectory of the bullet is clearly recognized. This clinical case reporting of a gunshot wound without evident entry hole, allows to discuss the diagnostic and therapeutic implications in the management of gunshot wounds cases with atypical entry and/or exit holes.

## Background

In case of abdominal gunshot wound, exploratory surgery has always been deemed indicated, as the likelihood that the bullet has caused a perforation of the gastrointestinal tract is high; unlike the lesions of solid organs, characterized by haemoperitoneum easily detectable by CT scan, intestinal perforation may not be immediately recognized, especially when the CT is carried out quickly, so that the air contained in the bowel has not the time to go outside and to be recorded as an indirect sign of perforation. However some Authors have recently proposed a more selective indication for surgery, based on serial CTs, in cases where the first CT is negative and the patient is haemodinamically stable, with the aim of avoiding unnecessary laparotomies. Two recently published papers highlight the role of laparoscopy in non-operative management of abdominal gunshot wounds, expecially when clinic is not clear or in presence of peritoneal signs [1, 2].

Obviously a quite different problem arises when entry and/or exit holes of the bullets are not clearly detectable, as highlighted by the reported singular case of unrecognized gunshot entrance hole, due to the fact that the patient was shot through his anus. To our best knowledge in scientific Literature there is any case of undetected entrance hole due to trans-anal gunshot, even if we found out 2 rare cases of unrecognized gastrointestinal bullet embolism [3–5]. We think that the reported case could be helpful to better understand the problem of unrecognized abdominal injuries following traumas.

## Case presentation

**Fig. 1:**
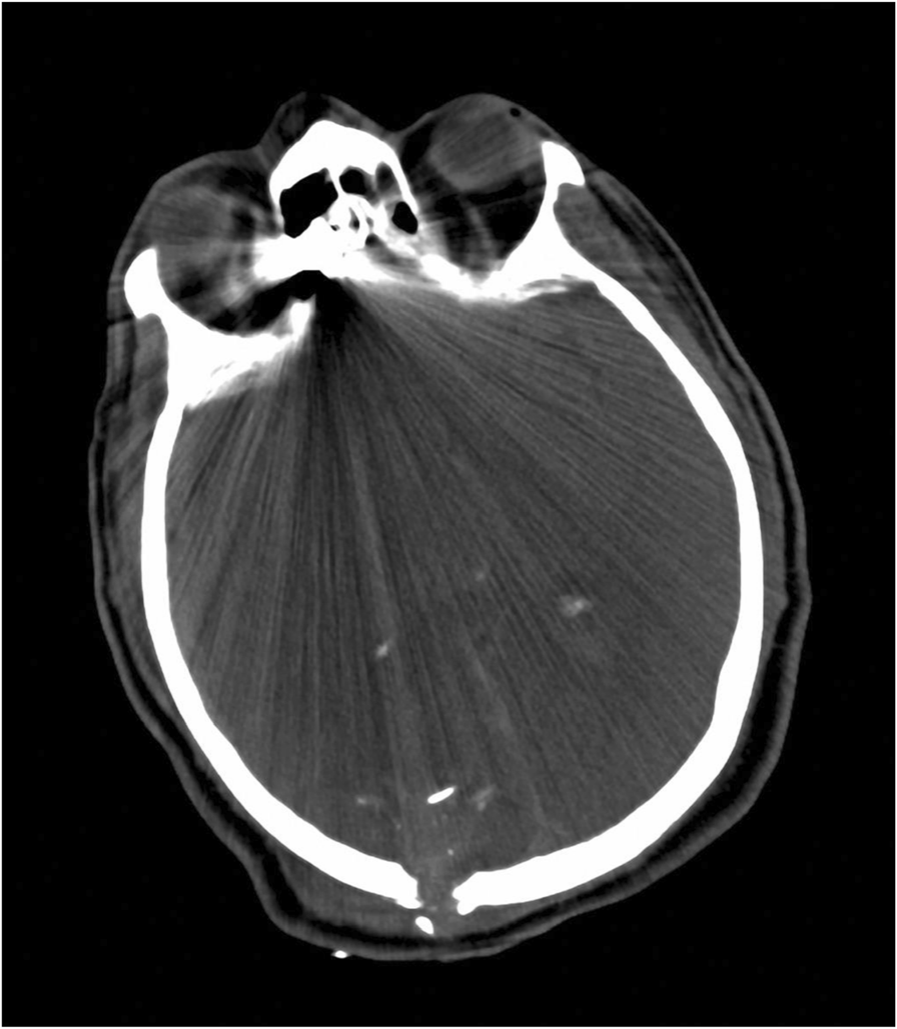
Retained bullet in the brain

**Fig. 2:**
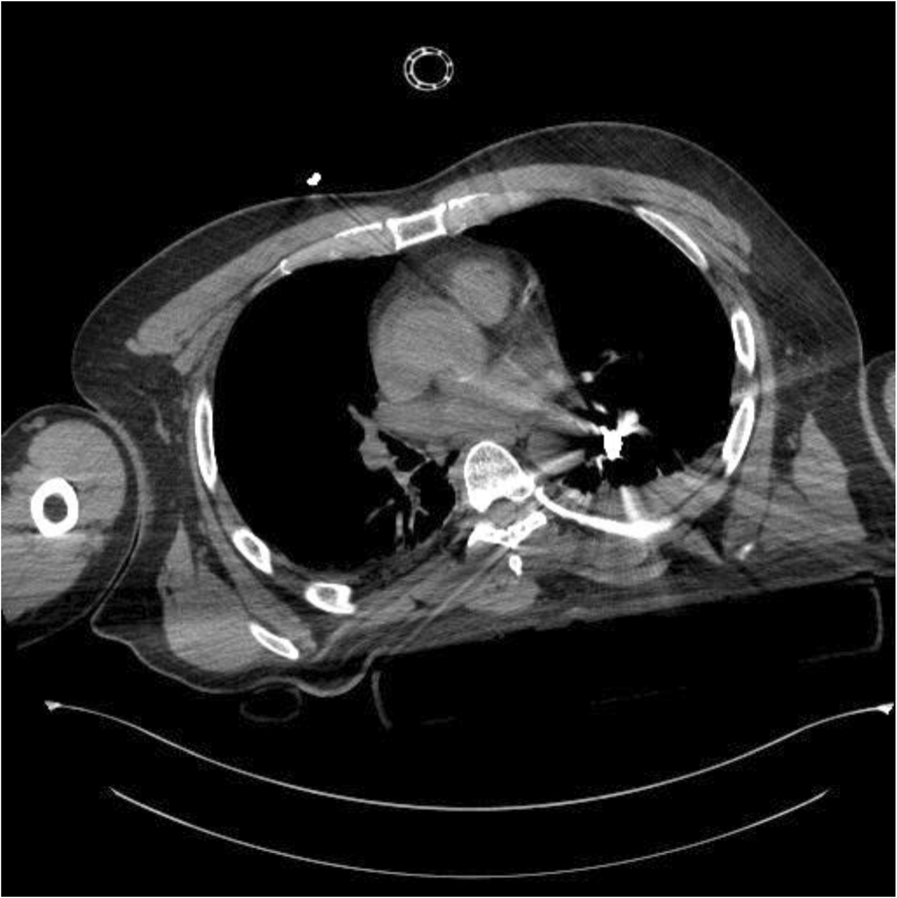
Retained projectile in the left lung

**Fig. 3:**
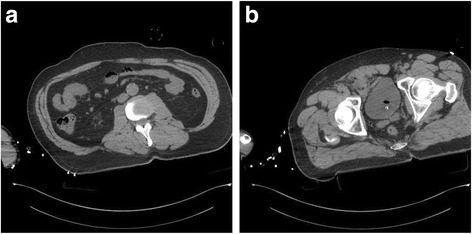
**a** Absence of free air outside the bowel. **b** Little air bubbles near to the pubis

The Patient, a 60-years old Caucasian male found unconscious in a trailer park of gypsies, was carried to Emergency Department at night, intubated and haemodynamically stable. At physical examination he presented a head gunshot wound, with an only visible bullet-hole located at the back of the neck. No other lesions were found. The total body contrast-enhanced CT scan performed at the Emergency Department revealed a large subdural haematoma, a retained bullet in the brain (Fig. 1) and another retained projectile in the left lung (Fig. 2), without any evidence of thoracic wall wounds; there was no air outside the bowel (Fig. 3a) even if little air bubbles could be recognized near to the pubis (Fig. 3b). These findings were difficult to be interpreted, both by the radiologist, the surgeon and the anaesthesiologist, as patient’s examination performed in the shock room after the CT scan confirmed the only presence of a single bullet-hole located at the back of the neck. As the Patient had no clinical and radiological signs of bowel perforation, it was decided for a “wait-and-see” attitude, scheduling a second CT scan for the following day.

**Fig. 4:**
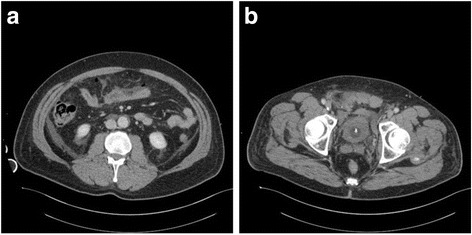
**a** Presence of peritoneal air bubbles outside the bowel. **b** Hematoma near the pubis

**Fig. 5:**
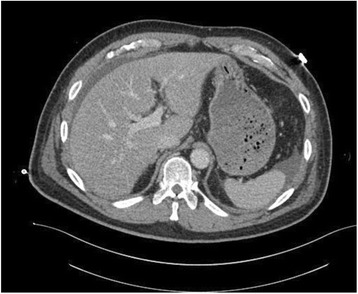
Peritoneal free fluid

About 14 h later the second CT scan showed the presence of hematoma near the pubis, peritoneal air bubbles outside the bowel (Fig. 4a, b) and diffuse peritoneal free fluid (Fig. 5). These findings were suspected for bowel perforation, thus the patient was translated into operative room: by laparotomy, the haemoperitoneum was drained and multiple perforations of small bowel were identified, associated with a single perforation of the upper part of the rectum, a double perforation of the stomach and a small hole in the left diaphragm, throughout which the bullet had reached the left lung. Multiple sutures of the rectum, the bowel, the stomach and the diaphragm and a small bowel resection were performed; the bulled retained in the lung was not reached nor removed. The only possible explanation for the trajectory of the bullet was that it was shot through the anus; evidently, after the blow on the head, the patient had fallen face down and the assailant shot him throughout the anus. This interpretation was later confirmed by aggressor interrogation by the Police.

The following course of the patient was uneventful; he was estubated after 3 days, having a Glasgow coma scale of 11, and he was discharged to rehabilitation 13 days after the incident; 4 months later the head bullet was removed by anterior approach, while the lung bullet was left in place.

## Discussion

From the analysis of this clinical report we concluded that the patient received two gunshot: the first was the only one that could be initially detected and it was fired to the back of the neck, while the second was fired directly throughout the anus; it perforated the rectum, and then it crossed the abdomen causing intestinal, gastric and diaphragmatic perforations, arresting into the left lung. It was not possible to make a correct diagnosis at first observation because a second bullet-hole could not be found.

To our best knowledge no similar cases have been reported in Literature. We only find 2 rare cases of unrecognized bullet embolism in the gastrointestinal tract causing colonic perforation [3, 4]. In both cases the authors claimed for the importance of accurate patient’s examination, both clinical and radiological in order to avoid delayed diagnosis of visceral injuries. In a paper of Apfelbaum et al. [5] the authors highlight the importance of correctly recognizing entrance and exit wounds in the Emergency Department and they admit that it could sometimes be difficult to discriminate the correct type of wound by surgeons. However the authors do not consider about the possibility of unrecognized gunshot wounds due to trans-anal or other natural orifices gunshots.

Many problems in the interpretation and reconstruction of the trajectory of the bullets have been reported in the Literature [6, 7]; a high index of suspicion should drive the diagnostic work-up, based on knowledge of ballistics and wounding potential of firearms; the goal is to anticipate the severity of a wound and detect earlier occult but severe internal lesions [8, 9]. In many cases, site of entrance and direction of bullet path could help the clinician to determine the potential of severe internal lesion. However, several singular cases have been described. The most frequent situation are atypical gunshot entrance holes, the wandering bullets, the spent bullet aspiration into the trachea-bronchial tree, and the so called “tandem bullet”, in which there are 1 only entry point and more than one exit point, meaning tandem projectiles or multiple projectiles entering through the same hole. Kuy et al. report of undetected urinary tract injuries due to a gunshot wound to the buttock and they also review other similar cases of undetected urinary injuries following gunshot [10–17]. Another atypical gunshot wound is the one reported from Ro et al., where the patient was shot from his left knee and the bullet travelled subcutaneously until the abdominal cavity perforating the left colon [18]. The authors highlight the importance of CT scan to accurate evaluation of bullet trajectory, however in this case the trauma team is helped by the presence of well evident entrance hole.

To our knowledge, this is the first report of the absence of an evident entry wound; since the CT scan showed the presence of a retained bullet in the left lung, the search for an entrance hole had been conducted with great attention by the surgeon, the anesthesiologist and the radiologist together. One possible explanation that was proposed was that the bullet had been swallowed and inhaled and then it had taken the path of a segmental bronchu. A second possible explanation was that the pulmonary bullet was fired many times later, during another gunshot assault. Retrospectively reassessing the information available after the first CT scan, the presence of air bubbles and small bone fragments in the extraperitoneal pelvis should have lead to a rectal examination. This was a major missing in the management of the here reported clinical case, even if the absence of indirect signs of bowel perforation was on contrast with the suspicion that the projectile had been shot from the anus and it reached the lung crossing the abdomen.

Performing the rectal examination could have anticipated the understaning of the sequence of the events, but probably the patients management would have not been different, due to his clinical stability and in absence of clinical signs of bowel perforation.

So the decision to repeat the CT scan after a few hours in order to monitor the presence of any sign of possible damage in the peritoneal cavity, was correct as allowed the diagnosis of the intestinal injures before the onset of clinical symptoms, avoiding the patient to die for septic shock [5, 6]. As an alternative, we could perform a diagnostic laparoscopy, as suggested by newer evidences for abdominal gunshot traumas with clinical suspicion of peritonitis or doubtful clinical signs [1, 2]; however, once again, our non-operative decision was supported by the stability of the patients, the absence of signs of bowel perforation at the first CT scan and the absence of peritoneal signs.

## Conclusion

Firearm wounds are increasingly observed in Emergency Department even in areas with classically low incidence of urban warfare, especially in Europe; they often raise new problems in interpretation both for denial by patients of firearm involvement [6] and for atypical clinical presentation [7]. The possibility of gunshot fired throughout natural orifices, such as anus, should be always taken into account in case of retained bullet without clear entry hole and, more important, the members of the trauma team should always keep in mind that a digital rectal exploration is mandatory in cases of abdominal gunshots wounds with unclear dynamics.

## Consent

Written informed consent was obtained from the patient for publication of this Case report and any accompanying images. A copy of the written consent is available for review by the Editor-in-Chief of this journal.

## Abbreviations

CT: Computed tomography
NOTES: Natural orifice transluminal endoscopic surgery
